# A Move in the Right Direction

**Published:** 1861-06

**Authors:** 


*A Move in the Right Direction.*—The New York Academy of Medicine have passed resolutions, declaring it derogatory to the dignity of the profession to publish cases and surgical operations in the daily papers, or to *suffer such publications to be made*—such proceedings being the ordinary practice of empirics. They are also forbid the publication of the transactions of the academy in any other than a strictly medical journal. The practice of inviting newspaper reporters to be present at surgical operations—which has occasionally occurred in this city—for the purpose of securing "the puff direct," is equally unprofessional, and should receive, as it justly deserves, the unqualified condemnation of every respectable physician. We must, however, do the justice to state that this practice has never been resorted to, or permitted, by the better class of our surgeons.

